# Bridging the digital divide for vulnerable patient telehealth

**DOI:** 10.1093/geroni/igaa057.3404

**Published:** 2020-12-16

**Authors:** Diab Ali, Monica Gillie, Kathy Jo Carstarphen

**Affiliations:** 1 University of Queensland - Ochsner Clinical School, New Orleans, Louisiana, United States; 2 Ochsner Health, New Orleans, Louisiana, United States

## Abstract

In New Orleans, Louisiana, 61% of advanced age patients have low health literacy, 50% report financial insecurity, and more than one-third have high hospital readmission rates and medical complexity scores. This population has been disproportionately impacted by the COVID-19 pandemic and the system-wide transition to remote, virtual visits. While COVID-19 has accelerated needs for impactful remote care and research, this has been impeded by low telehealth literacy and structural barriers, such as lack of internet and devices. There are major gaps in the literature regarding telemedicine services in geriatric and complex populations and efficacy rates are variable in these populations. There is currently no telehealth literacy screening tool designed for identifying patients with barriers requiring additional interventions to succeed, no uniform understanding of the factors affecting use or how to increase engagement, and telehealth models requiring fluent use of technology for older adults have been met with poor rates of completed visits and associated harm. Following a dedicated 358.5 hours to training 309 geriatric, complex patients in telemedicine using our pre-existing telehealth model, averaging 4.78 hours per patient, only 18.8% of these patients were subsequently able to connect to the provider virtually. Here we describe lessons learned and tools developed from the Ochsner MedVantage Network Innovation project, including the development of a telehealth literacy screening tool, the adaptation and provision of simplified, user-friendly tablets, and a randomized control trial to determine if increased accessibility to telehealth leads to improved healthcare outcomes, such as decreased hospital admissions and emergency department utilization.

